# Coupling of sensorimotor and cognitive functions in middle- and late adulthood

**DOI:** 10.3389/fnins.2022.1049639

**Published:** 2022-12-01

**Authors:** Astrid van Wieringen, Mira Van Wilderode, Nathan Van Humbeeck, Ralf Krampe

**Affiliations:** ^1^Research Group Experimental Oto-Rhino-Laryngology, Department of Neurosciences, KU Leuven, Leuven, Belgium; ^2^Research Group Brain and Cognition, Faculty of Psychology and Educational Sciences, KU Leuven, Leuven, Belgium

**Keywords:** listening in noise, postural control, functional mobility, processing speed, cognitive control, healthy aging

## Abstract

**Introduction:**

The present study explored age effects and the coupling of sensorimotor and cognitive functions in a stratified sample of 96 middle-aged and older adults (age 45-86 years) with no indication of mild cognitive decline. In our sensorimotor tasks, we had an emphasis on listening in noise and postural control, but we also assessed functional mobility and tactile sensitivity.

**Methods:**

Our cognitive measures comprised processing speed and assessments of core cognitive control processes (executive functions), notably inhibition, task switching, and working memory updating. We explored whether our measures of sensorimotor functioning mediated age differences in cognitive variables and compared their effect to processing speed. Subsequently, we examined whether individuals who had poorer (or better) than median cognitive performance for their age group also performed relatively poorer (or better) on sensorimotor tasks. Moreover, we examined whether the link between cognitive and sensorimotor functions becomes more pronounced in older age groups.

**Results:**

Except for tactile sensitivity, we observed substantial age-related differences in all sensorimotor and cognitive variables from middle age onward. Processing speed and functional mobility were reliable mediators of age in task switching and inhibitory control. Regarding coupling between sensorimotor and cognition, we observed that individuals with poor cognitive control do not necessarily have poor listening in noise skills or poor postural control.

**Discussion:**

As most conditions do not show an interdependency between sensorimotor and cognitive performance, other domain-specific factors that were not accounted for must also play a role. These need to be researched in order to gain a better understanding of how rehabilitation may impact cognitive functioning in aging persons.

## Introduction

Currently, more than 1 billion people in the world are 60 years and older^1^. A widespread sensory impairment in this rapidly aging population is age-related hearing impairment (ARHI or presbycusis). Hearing impairment is the third leading cause of disability for people ≥ 70 years, and the largest potentially modifiable risk factor for dementia (Livingston et al., 2020). Similarly, dramatic age-related differences occur for postural control, a phenomenon that is amply documented by the increased number of falls in the older population (Fuller, 2000). Global estimates suggest that 28–35% of people over 65 fall at least once a year. This estimate rises to 32–42% in people over 70 years of age^2^. Other sensorimotor functions affected by considerable age-related differences are tactile sensitivity and walking. Common to these different modalities is the trajectory of change: initial declines emerge during middle adulthood and accelerate after the 7th decade of life. Similar age-related changes also apply to cognitive processes like overall processing speed, fluid intelligence, or cognitive control processes (Baltes et al., 1999). The similarities of trajectories across functions and the considerable shared age-related variance in cross-sectional studies have motivated different theoretical accounts, arguing that the observed correlations reflect genuine couplings of sensorimotor and cognitive functions in their adult development.

### Coupling between cognitive and sensorimotor functions

Several theoretical accounts have linked age-related differences in sensory, sensorimotor, and cognitive functions (Verhaeghen and Cerella, 2002; Uchida et al., 2019), all of which depart from observations of correlations or shared age-related variance in older samples. Following earlier review papers, we distinguish between cascade models, common cause hypotheses, and compensation models (Li and Lindenberger, 2002; Kiely and Anstey, 2015; Humes and Young, 2016). Upward cascade models assume that degraded or reduced sensory information causes gradual declines in central cognitive functions. For example, age-related hearing loss may disturb the comprehension of spoken conversations and result in social isolation and reduced challenges for cognitive functioning. Reverse cascades have also been proposed in which declining central cognitive processing impairs sensory functions. For example, reduced inhibitory functions limit sustained attention necessary to identify sound sources and disambiguate auditory input. Specific versions of cascade models like the *perceptual degradation* and the *cognitive permeation* hypotheses emphasize that poor peripheral processing not only impairs higher-level cognitive processing, but that low-fidelity sensory inputs require more cognitive resources related to attention and executive functions for further processing. As a result, cognitive resources may not be available for their original purpose, which impinges on higher-level processing. An important implication of upward cascade models is that cognitive impairment should be reduced if the source of sensory malfunction is remedied or compensated for by, for example, cataract removal or a hearing aid. Positive evidence along these lines is far from equivocal(for reviews, see Schneider and Pichora-Fuller, 2000; Kiely and Anstey, 2015).

Common-cause explanations are resource accounts of behavioral aging, which assume that age-related differences within and across domains reflect, in part, a common set of senescent alterations (Lindenberger and Ghisletta, 2009). The differences between common-cause accounts refer to the type of central resource that is postulated and the causes of its deterioration in later adulthood. Precursors of modern common-cause theories emphasized the role of processing speed and its general age-related slowing (Cerella, 1985; Myerson et al., 1990; Salthouse, 1996). Slowing itself was conceptually linked to age-related changes in the brain, for example, impaired quality of axonic myeline and its presumed effects on signal conduction. The key idea was that processing speed, as assessed by, for example, simple reaction time or the digit-symbol substitution test from the WAIS, mediates age differences in cognitive functioning, including non-speeded tasks. Evidence for this “speed-mediation of cognitive aging” hypothesis in later adulthood was provided by Lindenberger et al. (1993) using the first wave sample from the Berlin Aging Study (BASE, age range 70-103 years). The authors found that speed fully accounted for common and specific age-related variances in reasoning, memory, knowledge, and fluency. Later, Lindenberger and Baltes (1994) included sensory (hearing and vision) and sensorimotor (balance-gait) variables in their mediation analyses of the same sample and found that vision and hearing together accounted for 93.1% of the age-related variance in the five intelligence factors in BASE. This includes the four factors mentioned above, but also the speed factor itself. Balance-gait added another 4.7% of the variance to the sensory variables and turned out to be as effective a predictor of age-related differences in intellectual functions as vision and hearing. Lindenberger and Baltes (1994) argued that age differences in intellectual and cognitive functions are the outcome of a third common factor or ensemble of factors that they attributed to age-related differences in the physiological state of the brain. Thus, unlike cascade models, common-cause accounts refrain from postulating a temporal order of age-related differences.

The cognitive compensation hypothesis proposed by Li and Lindenberger (2002) assumes that the aging brain tries to compensate for declines in sensorimotor functions by permanently recruiting cognitive resources (Li et al., 2001; Li and Lindenberger, 2002). This account is motivated by compensation accounts in neuropsychology (Cabeza et al., 1997; Reuter-Lorenz et al., 2000), and it also accommodates ideas from cascade models like the cognitive permeation hypothesis described earlier. An important difference with the latter approach is that the diversion of cognitive resources from their original purposes is seen as permanent. Li and Lindenberger based their proposition on the same correlational evidence as the common cause hypothesis; however, they also considered experimental evidence from two relevant approaches, notably simulations of auditory and visual decline and dual-task studies combining cognitive and sensorimotor tasks.

Most authors agree that the different accounts are not mutually exclusive and that a combination of mechanisms contributes to the coupling of cognitive and sensorimotor functions. For the described example of age-related hearing loss, one might imagine that peripheral damage causes reduced sociability resulting in central processing declines, accelerating listening difficulties or adapting to the handicap. Li and Lindenberger (2002) argued that a combination of common cause and compensation accounts would provide the best account of various findings. This argument is plausible from the perspective that (general) deterioration of functions precedes and triggers compensation. A second implication is that accelerated decline at advanced ages heightens the need for compensation, leading to even stronger correlations between sensorimotor and cognitive functions. While theories differ in the causal mechanisms or the direction of causality they emphasize, most models agree that the link between cognitive and sensorimotor functions becomes stronger with the advancing ages of the individuals (cf. reviews Boisgontier et al., 2013; Johannsen et al., 2022).

### Sensorimotor and cognitive functions with age

Loss of hearing sensitivity, often captured by pure tone audiometry, only partially explains difficulties in speech understanding. Damage to the inner ear also leads to distortion of (incoming) sound (e.g., Plomp, 1978) and loss of spectral and temporal resolution (e.g., Moore et al., 2012). Aging affects structures across the central auditory pathway (Profant et al., 2020) due to the reduction of neurons and inhibitory neural transmitters (Gao and Wehr, 2015; Jayakody et al., 2018). The loss of neural fibers, also caused by deterioration of ribbon synapses (“cochlear synaptopathy” Kujawa and Liberman, 2015; Parthasarathy and Kujawa, 2018), has consequences for listening in noise. Even without hearing impairment speech perception in noise declines by middle age (Goossens et al., 2017). The degrading effect of age is mediated by deficiencies in temporal processing and cognitive control and is also observed in persons without indication of cognitive decline. Aging as well as hearing impairment affect the neural encoding of speech cues in both subcortical and cortical structures (Anderson et al., 2012, 2021; Presacco et al., 2016; Goossens et al., 2018a,b), and these deficits in central auditory temporal processing have consequences for binaural processing (Vercammen et al., 2018b; Koerner et al., 2020), and the ability to separate a target speech message from a competing speech message (e.g., Helfer and Freyman, 2008). Given the abovementioned we wished to capture listening difficulties with a measure of speech understanding instead of the (predominantly) peripheral pure tone measure.

Aging also affects our ability to acquire and maintain a stable state of balance. Changes in the proprioceptive, visual and vestibular systems reduce the peripheral sensory reliability. In addition, postural control is constrained by central changes such as the reduction of white and gray matter integrity, affecting multisensory integration and motor execution at a (supra)spinal level. Together these developments negatively impact our sense of body position and coordination (Michalska et al., 2021). Measurements of postural control during upright stance are frequently recorded with a force plate which registers fluctuations in the participant’s center of pressure (COP) across time. Conventionally, these fluctuations are quantified using the total displacement or the area in which COP movement occurs. Using these metrics, Abrahamová and Hlavacka (2008) showed substantial age-related differences in performance starting around 60 years of age.

Even though these metrics are sensitive enough to address age-related differences in sway behavior, more elaborate methods have gained popularity because of their potential to address the neuromuscular mechanisms underlying postural control (Lacour et al., 2008). Based on Einstein’s theory of Brownian motion, the stabilogram diffusion analysis (SDA) analyzes mean squared C0P displacement at different timescales. The short timescale behavior reflects an open-loop control scheme tempering the inherently unstable body. Once a critical threshold is reached, long-term closed-loop mechanisms come into effect resulting in anti-persistent corrective feedback motion (Collins and De Luca, 1993). Previous age-comparative studies applying the SDA have consistently found pronounced increases in short timescale displacement with age (Collins and De Luca, 1995; Laughton et al., 2003; Norris et al., 2005). A common explanation for this is the elevated level of muscle activity found in older age (Laughton et al., 2003; Finley et al., 2012). Some authors argue that these processes induce a shift, going from automatic to more cognitive processing of movement (Heuninckx et al., 2008, 2010; Goble et al., 2010).

Mobility tests are essential to assess function and ambulation in a frail elderly population (Butler et al., 2009). While standard medical examinations aim to screen and diagnose diseases/injuries, they do not provide sufficient info regarding the patient’s daily living capabilities (Tinetti et al., 1988). Functional mobility is a person’s ability to move around safely and independently while accomplishing everyday activities (Bouça-Machado et al., 2020). These activities include basic mobility skills such as rising from a chair, walking, turning and bending over and are significant predictors for falls, ongoing disability, and nursing home admission (Guralnik et al., 1994). Multiple tests have been designed to assess functional mobility, including self-reported questionnaires and laboratory-based assessments. The timed up-and-go test (TUG) provides an easy-to-use alternative showing reliable results that correlate highly with other gold-standard assessments such as the Barthel index and the Berg Balance Scale (Podsiadlo and Richardson, 1991). Studies have shown a moderate correlation between TUG and age (Khant et al., 2018). Additionally, age can predict TUG performance even when cognitive status is controlled for (Ibrahim et al., 2017).

Among other modifications, a declining receptor density and skin elasticity reduce our capacity to perceive touch pressure and vibration (Stevens et al., 2003; Wells et al., 2003). This is encompassed by significant changes in brain recruitment, mainly reflected by over-activation of the somatosensory network to compensate for impaired brain functions (Brodoehl et al., 2013). Perry (2006) investigated tactile age-related alterations in plantar sensitivity and found pronounced differences between young and older adults from the seventh decade onward.

Studies investigating common causes for age-related differences in cognitive and sensorimotor functions typically used measures from IQ tests, emphasizing latent constructs for processing speed of fluid intelligence. In the present study, we took a different approach. Although we included a measure of general processing speed, we focused on the three core cognitive control functions, working memory/updating, inhibitory control, and cognitive flexibility (Miyake and Friedman, 2012). While many multisensory integration processes occur automatically in association cortices, cognitive control processes involve frontal lobe circuitry (D’Esposito and Postle, 2015; Gerver et al., 2020), which is most sensitive to aging. The following paragraphs summarize age-related differences in our cognitive measures and discuss how sensorimotor processes draw on processing speed, working memory, inhibitory control, and cognitive flexibility.

Older adults need more time to process information in the same tasks than younger ones (Salthouse, 1996, 2009, 2019), and this has consequences for the comprehension and recall of speech (Wingfield, 1996, Gordon-Salant and Fitzgibbons, 1999; Goy et al., 2013) and temporal processing, such as the detection of gaps and binaural hearing (Strouse et al., 1998, Füllgrabe et al., 2015). Age-related differences in processing speed also significantly affect gait speed (Soumaré et al., 2009; Lowry et al., 2012, Desjardins-Crépeau et al., 2014; Killane et al., 2014) and mobility (Rosano et al., 2005).

Working memory (WM) is defined as a limited-capacity system by which we store, process, and manipulate information. Crucial functions are updating, replacing stored information with new incoming information, and maintaining the stored information in memory (Gajewski et al., 2018). Listening, especially in noise, draws heavily on working memory. When the peripheral and/or central encoding of speech sounds is distorted, a listener relies on implicit (or automatic) and explicit cognitive processing mechanisms to enable a fast retrieval from memory or knowledge to fill in the missing information, ignore the irrelevant noise and selectively focus attention on the spoken message (e.g., Rönnberg et al., 2013). While hearing impairment seems to be the main factor underlying speech perception problems in background noises, age explains a significant part of the communicative impairment (Gordon-Salant and Cole, 2016). WM is highly influenced by age, and reduced WM capacity makes a person more susceptible to reverberation and echoes (Reinhart and Souza, 2016). Age-related cognitive decline is also a leading cause of the decline in motor performance (Krampe, 2002; Li and Lindenberger, 2002). Behaviorally, performance in both the working memory and motor task decline with increasing task difficulty (Lindenberger et al., 2000; Li et al., 2001; for a review Yogev-Seligmann et al., 2008), although resource allocation is flexible and can change over the lifespan to compensate for age-related decline in sensorimotor and cognitive processing.

Inhibition, the ability to suppress irrelevant information (Miyake et al., 2000), is also susceptible to aging and, consequently, affects different sensory and sensorimotor functions. As poor inhibition increases susceptibility to background noise (Janse, 2012), persons with poor inhibition will find it increasingly difficult to understand speech in noise as noise increases (Knight and Heinrich, 2017). For instance, older adults are more influenced by the semantic content of a to be ignored voice when different persons are speaking than younger adults (Tun et al., 2002).

Suppressing irrelevant information is also crucial for postural control/mobility/balance (see Kwag and Zijlstra, 2022 for a recent scoping review). Mirelman et al. (2012) report that executive functioning, including inhibition, predicted falls over the five years following cognitive assessment. A more recent study shows that participants who are better at inhibiting their responses in the stop signal task were better at inhibiting an unwanted leg response than grasping a supportive handle (England et al., 2021).

Cognitive flexibility, the ability to switch between tasks or mental sets (Kray and Lindenberger, 2000), is crucial for listening, whether needed to monitor multiple simultaneous voices (Kidd et al., 2005), to focus auditory spatial attention (Singh et al., 2013), to process unattended speech (Perrone-Bertolotti et al., 2017). Whether task switching is compromised in healthy aging remains somewhat unclear because of its interdependence with inhibitory control. Using a binaural-listening paradigm, Oberem et al. (2017) studied age-related differences in the ability to intentionally switch auditory selective attention between two speakers. Significantly higher reaction times and error rates were observed for older participants than for younger ones. Previously, Lawo and Koch (2014) also reported that the ability to switch auditory attention in a selective listening task intentionally does not seem to be compromised in healthy aging. Mobility also taxes cognitive flexibility. For example, faster individuals during the timed-up-and-go task demonstrate better cognitive flexibility (Berryman et al., 2013). An increased congruency effect when standing compared to sitting is observed in an auditory cue task-switching paradigm with different postural control demands (Stephan et al., 2018).

### Outline of current study

Over and beyond the expected age-related differences in sensorimotor and cognitive functioning, this study examined whether age-related individual differences in processing speed and cognitive control processes (working memory, inhibition, task switching) were coupled with differences in performance in sensorimotor processes during middle- and late adulthood. Our study design was guided by four considerations that differ from most of the earlier work. First, we deviate from earlier research which used psychometric intelligence tests as a general measure of cognitive ability. Instead, we chose for core cognitive control measures, notably inhibition, working memory updating and switching to determine which candidate mechanisms drive the age-related coupling of sensory and cognitive functions. In addition, we included processing speed, which is closely related but not identical to fluid intelligence. Second, we focused on listening in noise, postural control, functional mobility and tactile sensitivity on the sensorimotor side, as these functions become more challenging with age and have not been researched before in the same population. Hearing ability has been assessed by the ‘pure tone average’ in previous studies. We argue that a measure of speech understanding in noise is better suited to capture the influence of cognitive processes and also much closer to the ecological reality of aging listeners. Third, we targeted the transition between middle- and late adulthood to pick up early and potentially subtle changes in sensorimotor and cognitive skills. From perspectives of prevention and intervention this is also the most critical period to investigate. Fourth, we exclusively tested individuals who had passed cognitive screening to minimize confounding effects of accelerated cognitive decline.

Following the reasoning of common-cause accounts, we first asked whether processing speed or our measures of sensorimotor performance mediated age effects in the three cognitive control variables. We then asked whether individuals who had poorer (or better) than median cognitive performance for their age group also performed relatively poorer (or better) on sensorimotor tasks. Our driving hypotheses were that individuals with poor processing speed, inhibitory control, cognitive flexibility, and working memory updating also perform relatively poorly on the sensory and sensorimotor measures. We expected the coupling to emerge during middle age and to intensify in later adulthood.

## Materials and methods

### Participants

Four age cohorts, two middle-aged and two older-age, were defined, i.e., (46-55), (56-65), (66-75), and (76-86). A hundred and twelve healthy participants were recruited through campus, market advertisements, and e-mail. Prior to testing, a brief questionnaire assessed health-related issues, i.e., whether participants smoked, wore one or two hearing aid(s), had been hospitalized, had experienced falls within the last year, had a knee or hip prosthesis, had suffered chronic ear infections or undergone ear operations, had ever had physical therapy. Six participants, mainly in the two older categories, had hearing aids (5 bilateral, one unilateral), and six participants had experienced falls and wore knee or hip prostheses (see Supplementary material). All participants performed the modified version of the CODEX (Ziso and Larner, 2019). The Cognitive Disorders Examination or Codex is a 3-min test with high sensitivity and specificity for dementia diagnosis (Belmin et al., 2007). A participant is assigned 1 of 4 levels of the CODEX (A = very low, B = low, C = high, and D = very high probability of dementia). Only data of persons with scores A or B were included. Eight persons with scores of C or D were excluded, and eight women of the two youngest categories were randomly excluded to analyze an equal number of participants per age category (*n* = 24). No difference was observed between the A and B scores of the CODEX (χ^2^ (3) = 7.18, *p* = 0.066).

Table 1 lists the demographics of the remaining 96 participants, including educational level and estimated total IQ per group. A Kruskal-Wallis chi-square test yielded statistical significance for education level (ranked in years, (H(3):11.149, *p* = 0.01). *Post hoc* Dunn tests showed that only the 46-55 and the 66-75 age groups differed significantly from each other (*p* = 0.03). The total IQ was based on the sum of the scaled scores of the performance and verbal IQ according to the norm values of the Dutch version of the Wechsler Adult Intelligence Scale (Wechsler, 2001). Scaled scores for each domain were estimated based on the digit symbol substitution (performance IQ) and the digit span task (verbal IQ). All participants provided informed consent to the study, which the Medical Ethical Committee approved of KU Leuven/UZ Leuven. They received 11€ for participating.

**TABLE 1:** Sample characteristics.

				Gender		Education level				
Age	Mean age	Sd age	N	Male	Female	Low	Average	High	IQ	Sd IQ
46–55	51.8	2.29	24	9	15	1	2	21	118	16.2
56–65	61.6	3.05	24	12	12	1	4	19	119	20.3
66–75	71.3	2.37	24	12	12	2	11	11	116	20.2
76–86	80.8	3.54	24	12	12	5	5	14	104	19.0

Education: low = obligatory schooling not completed, average = obligatory schooling completed, high = higher education.

### Procedure

Each participant was tested individually at the lab or at home. Testing took, on average, 1.5 h and always started with listening in noise. All other tests were randomized. In addition to the tests mentioned below, we also performed a posture verbal fluency dual-task test, and we asked participants to fill out the 12-item Speech, Spatial and Qualities of hearing questionnaire (Noble et al., 2013). These data did not belong to the scope of this paper.

#### Listening in noise

Listening in noise was assessed with the Flemish version of the digits in noise test *(DiN)*. This paradigm has high sensitivity and specificity for detecting sensorineural hearing loss (Jansen et al., 2013; Smits et al., 2013). Three speech digits were presented in noise via a Samsung tab A tablet and calibrated Peltor H7A headphones to both ears (without hearing aids for the six persons mentioned in the Supplementary material). The level of the speech was fixed at 65 dB A, and the first triplet was presented at a –2 dB signal-to-noise ratio. Speech reception thresholds (SRT) in broadband noise were determined utilizing an adaptive procedure using triplet and digit scoring (Denys et al., 2019).

#### Postural control

In the static balance, postural control task, participants are asked to stand as still as possible for 30 seconds on a Nintendo^®^ Wii Balance Board (Nintendo, Kyoto, Japan) while looking at a black dot placed 1 meter in front of them at the eye level. Feet are positioned parallel close to each other in the center of the board, with the toes pointed forward. At the start of each trial, the instructor provides a cueing signal “ready” and a starting signal “start” when the time measurement commences. After a warm-up trial, four test trials are assessed. For each trial, the center of pressure (COP) is calculated based on four load sensors positioned at the corners of the Wii balance board using a bluetooth connected computer with CU BrainBLoX software (Cooper et al., 2014). This data is then linearly interpolated to a 100 Hz frequency and low-pass filtered with a fourth-order 13 Hz Butterworth filter using a custom-written script in R (R Core Team, 2021). Evaluation of postural control included the stabilogram diffusion analysis (SDA) proposed by Collins and De Luca (1993). The SDA is based on Einstein’s theory of Brownian motion and analyzes mean squared CoP displacement at different timescales. The short timescale behavior reflects an open-loop control scheme tempering the inherently unstable body. Once a critical threshold is reached, long-term closed-loop mechanisms result in anti-persistent corrective feedback motion. The slopes of the linear regressions fit on short- and long-timescale regions were used to quantify these mechanisms, i.e., the short-term diffusion coefficient and the long-term diffusion coefficient, respectively. Additionally, the critical time interval represents the time interval separating both regions.

#### Functional mobility: Timed up and go

The ability to rise from a chair is a critical mobility component and was assessed with the timed-up-and-go (TUG, Podsiadlo and Richardson, 1991), a widely used clinical test and screening tool. Participants were instructed to rise from a chair, walk 3 m straight, turn around, walk back and return to the same sitting position as fast and safely as possible (Schoene et al., 2013). Running was not allowed, and the 3-m distance was indicated on the floor. After a warm-up trial, three test trials were conducted. At the start of each trial, the instructor presented a cueing signal “ready” and a starting signal “start,” after which the time measurement commenced. Timing stops when the participants’ shoulder blades touch the chair’s backrest (Vereeck et al., 2008). The outcome measure was the average time (s) required to finish the three test trials.

#### Tactile sensitivity

A monofilament test measures a participant’s cutaneous perceptual threshold by applying light touch pressure to the skin. This is often done with Von Frey filaments, i.e., 20 nylon filaments with ascending stimulus intensity. The filament must be placed perpendicular to the skin, and force is gradually increased until the filament bends, thereby regulating the stimulus intensity of a given filament. Participants were asked to sit in a chair and take off their right sock. A dot was placed lateral to the fifth metatarsophalangeal joint of the right foot. Pressure was applied to the dot with filaments of different intensities, and participants were instructed to close their eyes and answer with “Yes” or “No” when asked whether they felt something. An interlacing adaptive staircase method was used with staircases A, for odd-numbered stimuli, and B, for even-numbered stimuli. The task included 18 trials in staircase A, 17 trials in staircase B, and five-catch trials (Berquin et al., 2010). A negative response to a filament yielded a filament of higher intensity in the subsequent trial and vice versa. Each time the response within a staircase differed from the preceding response (turnaround point), the step size was adjusted according to the 4, 2, and 1 stepping algorithm (Dyck et al., 1993). A custom-written software was used to register the responses and indicate the stimulus intensity. The average stimulus intensity of the turnaround points with step size one was registered for each staircase. The average of these stimulus intensities served as an outcome measure.

#### Processing speed/Digit symbol substitution

The digit symbol substitution is a paper and pencil test (Wechsler, 2001) used to proxy processing speed (Jaeger, 2018). The test consisted of a key grid of digits and matching symbols. The participant was instructed to fill out the empty boxes with the symbol that matches each digit as quickly and accurately as possible. This task requires planning and strategizing, updating digit-symbol matches, and filtering out irrelevant information (e.g., symbols that may look alike). First, participants were instructed to fill all digit-symbol associations up to the bold black line. Once the practice section was completed and corrected if necessary, participants were asked to continue filling in the digit-symbol associations as fast as possible without skipping any. The score reflects the number of correct digit–symbol matches within 120s.

#### Working memory updating/2-back task

Given that auditory input is constantly changing in daily life, updating information is a critical component of speech understanding in noise (e.g., Sussman and Winkler, 2001). The 2-back task taps into working memory updating (Gajewski et al., 2018). Letters appear consecutively on a 17′ monitor for 300 ms. Participants were instructed to press the space bar if a letter was identical to the second-last letter. The task required updating incoming information. A computerized version was used (OpenSesame 3.1, Mathôt et al., 2012). First, participants performed a short warm-up trial of 20 letters in which oral feedback was provided. Later, a test trial of 156 letters was presented, including 20% target and 80% non-target letters. Each stimulus was presented for 300 ms and the interstimulus time was 1,400 ms. During that period, a response could be provided. The outcomes were the responses and the reaction times (RT) of the correct scores (hits). RTs less than 100 ms and more than 1,200 ms were scored as misses. Subsequently, d’ was determined from the responses for further analyses.

#### Inhibitory control/Stroop task

The Stroop task assesses inhibitory control (Scarpina and Tagini, 2017) by requiring participants to identify the color in which a symbol or word is presented while ignoring the word’s meaning. Two conditions were presented together via OpenSesame 3.1 (Mathôt et al., 2012): a neutral condition in which the letters contained four or five X’s or an incongruent condition in which the colored shape contained a written word, consisting of a written color that is different from the color in which the word was written. Participants responded with four keys on an external keyboard corresponding to the letters (f, k, d, j). The participant was asked to only respond to the color of the word/X’s. A practice trial was offered before testing with four neutral and eight incongruent trials. Participants were instructed to keep their fingers on the respective keys of the keyboard. The actual test contained 48 trials, of which half were neutral and half incongruent. Reaction times were registered. Preprocessing the data was according to Gajewski et al. (2020). The first trial was deleted, as well as all response times shorter than 100 ms and response times longer than 2 SD of the mean response time (per age group and condition). We used the inverse efficiency score (IES) for further analysis, reflecting an overall performance index while accounting for speed and accuracy trade-off. The IES was calculated by dividing the mean reaction times of the correct trials by the overall accuracy. Afterward, Stroop interference (MacLeod, 1991) was calculated by subtracting the IES in the neutral condition from the IES in the incongruent condition (SI = IES_incongruent_ – IES_neutral_).

#### Task switching/Color-shape switch task

Participants are asked to switch between two or more task sets in a task-switching paradigm. Performance on the color-shape switch task reflects global cognitive control, cognitive flexibility, and working memory (Sicard et al., 2020). The color-shape switch task is also administered with OpenSesame 3.1 (Mathôt et al., 2012). It consists of three non-verbal parts: in the first part, participants indicate the color of a shape (A), either blue or yellow, in a fixed (non-switch) block (*n* = 24, AA AA AA AA AA). In the second part, another fixed block is presented, namely the shape of the form, either round or square (B, *n* = 24, BB BB BB BB BB). In the third part, a mixed (switch) block is presented, and the participant must alternatively focus on the shape or color (*n* = 48, AA BB BB BB). For the latter condition, participants are instructed to focus twice on the shape, then twice on the color, and then twice on the shape. The background color (black/gray) is presented on the screen to indicate whether to focus on the shape or the color. Similar to the Stroop task, within each block, the first trial was deleted, as well as reaction times shorter than 100ms and reaction times longer than two SD of the mean response time. The general switch costs (also known as mixing costs) reflect the ability to maintain and select among different task sets in working memory (AA AA AA vs. AA BB AA). General switch costs are calculated by subtracting the IES of the fixed block from the IES of the mixed block. Specific switch costs are calculated by subtracting the IES of the repetition trials in the mixed block (AA or BB) from the switch trials in the mixed block condition (AB or BA).

#### Digit span

The digit span test was used to estimate verbal IQ (Table 1). This test was taken from the Wechsler Adult Intelligent Scale (Wechsler, 2008). A list of digits is presented verbally at a rate of one per second. The participant must either repeat the list in the same order (digit span forward, short-term memory) or the reverse order (digit span backward, working memory). All digits must be in the correct order for the list to be marked correct. The lists start at a length of two digits (maximum 8 for the forward digit span, maximum 7 for backward digit span), and two lists of each length are presented. The test is stopped when two lists of a certain number of digits are recalled incorrectly. The outcome is the number of correct sequences.

### Statistical analyses

Statistical analyses were conducted with R (R Core Team, 2021). Each variable was transformed to obtain normality using either a log transformation or a Box-Cox negative power transformation (Fox and Weisberg, 2019). This was done across age groups. The effects of age group on sensorimotor processes, processing speed, and cognitive control processes were analyzed using linear models (LMs). Three orthogonal age group contrast were specified *a priori*, comparing (a) the mean of the two middle-aged groups with the mean of the two older adult groups; (b) the two middle-aged groups with one another; and the two oldest groups against each other. Following the approaches by Lindenberger et al. (1993) and Lindenberger and Baltes (1994), we assessed the degree to which age-related variance in cognitive control measures was mediated by processing speed and the four sensorimotor functions. To this end, we performed a causal mediation analysis using the package Mediate in R (Tingley et al., 2014). The R package “Mediate” uses a non-parametric bootstrapping method to estimate the significance of the causal mediation effects in a linear model. Finally, we determined the coupling between cognitive and sensorimotor functions by applying median splits within each age group to identify individuals with high and low levels of performance. Median group was added as a fixed effect to the LM described earlier. *Post hoc* tests were performed through Bonferroni-corrected t-tests unless unequal variances were detected, in which case Bonferroni-corrected Welch t-tests were used.

## Results

The current study aimed to examine whether age-related individual differences in processing speed and cognitive control processes (working memory, inhibition, task switching) were coupled with differences in performance in sensorimotor processes during middle- and late adulthood. We present our results in three parts. We established age-related differences for sensorimotor and cognitive functions in the first part. We then applied two different approaches toward determining whether and how aging of sensorimotor and cognitive processes mutually constrain each other. First we walk on the trails of common cause hypotheses by assessing to what degree processing speed and sensorimotor functions mediate age-related variance in cognitive control measures. Second, we turned our perspective around by asking whether high and low performance levels in processing speed or cognitive control functions coincided with better or worse performances in sensorimotor tasks. In our analyses, we included gender as a fixed factor. Given that it did not improve the model fit significantly, gender was further excluded from the analyses reported below.

### Age-group differences in sensorimotor and cognitive functions

**FIGURE 1:**
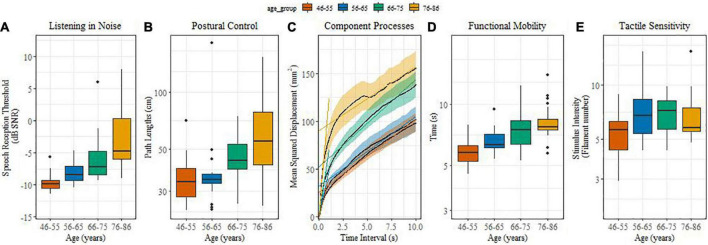
**(A–E)** (boxplots): Effect of age on sensory and sensorimotor processes. **(A)** Listening in noise in noise; **(B)** Postural control; **(C)** Component processes; **(D)** Functional mobility; **(E)** Tactile sensitivity.

Figures 1A-E illustrate age-related differences in sensory and sensorimotor functions. For listening in noise (Figure 1A), the younger groups performed systematically better than their older counterparts. This was reflected by reliable differences between middle-aged and older adults (β = 0.19, SE = 0.02, *t* = 9.02, *p* < 0.0001) and between the age groups of 46-55 and 56-65 (β = 0.05, SE = 0.01, *t* = 3.56, *p* = 0.001), 66-75 and 76-86 (β = 0.05, SE = 0.01, *t* = 3.67, *p* = 0.001). Speech in noise thresholds decreased, on average, by 0.15 dB SNR per annum.

Postural control performance showed reliable age effects for path length (Figure 1B). Middle-aged adults performed better compared with older adults (β = 0.32, SE = 0.064, *t* = 5.03, *p* < 0.0001), and the 66-75 age group performed better when compared with the 76-86 one (β = 0.11, SE = 0.04, *t* = 2.48, *p* = 0.0015). Figure 1C illustrates the component processes (SDA parameters) of postural control. Only the short-term diffusion coefficient, i.e., the early time-scale slope, yielded reliable age differences. Short-term diffusion coefficients of middle-aged adults were lower than those of older adults (β = 0.58, SE = 0.12, *t* = 4.99, *p* < 0.0001), while the long-term diffusion coefficient and the critical time interval did not differ significantly between age groups.

With increasing age significantly more time was needed for the timed-up-and-go test (Figure 1D). Functional mobility was significantly different between the middle aged and older adults (β = 0.03, SE = 0.003, *t* = 6.02, *p* = 0.0001), between 56-65 and 76-86 (β = 0.01, SE = 0.002, *t* = 2.24, *p* < 0.03), and between 46-55 and 56-65 year olds (β = 0.01, SE = 0.002, *t* = 3.17, *p* = 0.005).

Different from the other sensorimotor functions, our measure of tactile sensitivity turned out to be less sensitive to age (Figure 1E) and only showed a reliable difference between the middle-age groups of 46-55 and 56-65 (β = 0.11, SE = 0.03, *t* = 3.4, *p* = 0.001).

**FIGURE 2:**
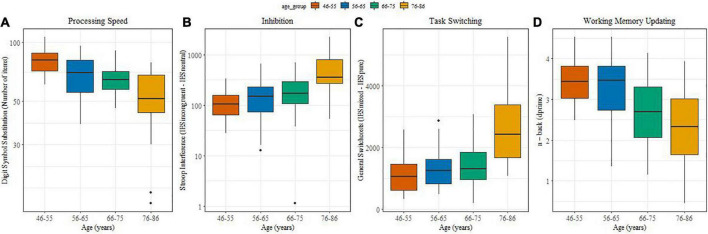
**(A–D)** (boxplots): Associations between age and performance on cognitive measures. **(A)** Processing speed; **(B)** Inhibition; **(C)** Task switching; **(D)** Working memory updating.

Figures 2A–D illustrate potential changes in processing speed, inhibitory control, task switching and working memory updating with age. Processing speed (Figure 2A) differed significantly between the two middle aged and the older groups (β = −33.25, SE = 0.35, *t* = 3.72, *p* < 0.0001), between the 66-75 and 76-86 group (β = −11,58, SE = 0.25, *t* = 3.29, *p* = 0.0065) and between 46-55 and 56-65-75 (β = −10,91, SE = 0.25, *t* = 1.26, *p* = 0.01). With advancing ages inhibiting information became more difficult. Stroop interference values (Figure 2B) increased significantly between the middle-aged groups and the older groups (β = 1.09, SE = 0.22, *t* = 4.84, *p* = 0.001 0.05), and between the 66-75 and 76-86 group (β = 0.53, SE = 0.15, *t* = 2.18, *p* = 0.00070.05).

Regarding cognitive flexibility, general switch costs also increased with age (Figure 2C) and they were significantly higher for older adults in comparison with middle-aged adults (β = 7.31, SE = 1.6, *t* = 4.57, *p* < 0.0001) as well as between the 66-75 and 76-86 groups (β = 5.95, SE = 1.13, *t* = 5.26, *p* < 0.0001). Variability in performance was pronounced in the oldest age group. Specific switch costs did not yield an effect of age (not shown).

Working memory updating (Figure 2D) showed a reliably lower d’ for older compared with middle-aged individuals (β = –1.55, SE = 0.33, *t* = −4.63, *p* < 0.00015).

### Processing speed and sensorimotor functions as mediators of age-related variance in cognition

We used causal mediation analysis to determine how much of the age-related variance (ARV) in three cognitive control measures was mediated by processing speed or sensorimotor functions. Results are shown in Table 2. The average causal mediation effect (ACME) reflects the mediation effect of age through processing speed or the sensorimotor measures. The average direct effect (ADE) reflects the direct effect of age on the cognitive control measures when controlled for the mediator. The total effect is the sum of the ADE and ACME, which reflects both the direct and indirect effect of age on the dependent variable (cognitive measures). The proportion mediated describes the proportion of age on the cognitive measure that passes through the mediator. Our results show a reliable mediation effect of age through processing speed and functional mobility on task switching and inhibitory control, with proportions varying from 41% to 52%. None of the other variables show a reliable mediation effect.

**TABLE 2:** Causal mediation analysis.

Dependent variables	Task switching	Inhibitory control	Working memory updating
**Processing speed**			
Mediation effect of age effect through processing speed (ACME)	0.12***	0.01**	–0.01
Direct effect of age on the dependent variable when controlling for processing speed (ADE)	**0.11** *	**0.01** *	−0.03*
Total effect (ADE + ACME)	**0.24** ***	**0.02** *	−0.04*
Proportion mediated	**0.52** ***	0.41**	0.25
**Listening in noise**			
Mediation effect of age effect through listening in noise (ACME)	0.04	–0.004	–0.001
Direct effect of age on the dependent variable when controlling for listening in noise (ADE)	0.20**	0.02***	−0.04**
Total effect (ADE + ACME)	0.24***	0.02***	−0.04***
Proportion mediated	0.16	–0.23	0.04
**Functional mobility**			
Mediation effect of age effect through functional mobility (ACME)	0.09*	0.01***	–0.01
Direct effect of age on the dependent variable when controlling for functional mobility (ADE)	0.14*	0.01*	−0.03***
Total effect (ADE + ACME)	0.23***	0.02***	−0.04***
Proportion mediated	0.42*	0.41***	0.22
**Postural control**			
Mediation effect of age effect through postural control (ACME)	–0.01	–0.0001	–0.004
Direct effect of age on the dependent variable when controlling for postural control (ADE)	0.25***	0.02***	−0.04***
Total effect (ADE + ACME)	0.24***	0.02***	−0.04***
Proportion mediated	–0.04	–0.01	0.10

The table lists the ACME, ADE, total effect and the proportion mediated for each mediator (processing speed and the sensorimotor measures) and each dependent variable (task switching, inhibitory control and working memory updating). Education: low = obligatory schooling not completed, average = obligatory schooling completed, high = higher education. Statistically significant results are indicated in bold with an asterisk (*p < 0.05, **p < 0.01, ***p < 0.005).

### Coupling of cognitive and sensorimotor functioning: Median splits

As a final assessment of coupling between cognitive and sensorimotor functions, we performed median splits based on cognitive ability within each age group (processing speed and the three cognitive control variables). The high-low performance distinction, so derived, was used in the LM model with the three age-group contrasts as an additional predictor. Analyses were conducted separately for cognitive variables and three sensorimotor functions (listening in noise [DiN], functional mobility [TUG], and the postural data assessed through the short-term diffusion coefficient). The long-term diffusion coefficients and the critical time interval in posture tasks, specific switch costs and tactile data were not further analyzed because they did not show reliable age effects to begin with. Main effects related to the median-split factor indicated that individuals with low performance on a certain cognitive measure also differed reliably from high-performing individuals in the sensorimotor function in question. In other words, they point to a coupling of the cognitive and the sensorimotor function under consideration. Interactions between the three age-group contrasts and the median-split factor indicated that coupling strength (i.e., the differences in sensorimotor functioning between high- and low cognitively performing individuals) depended on age group.

**FIGURE 3:**
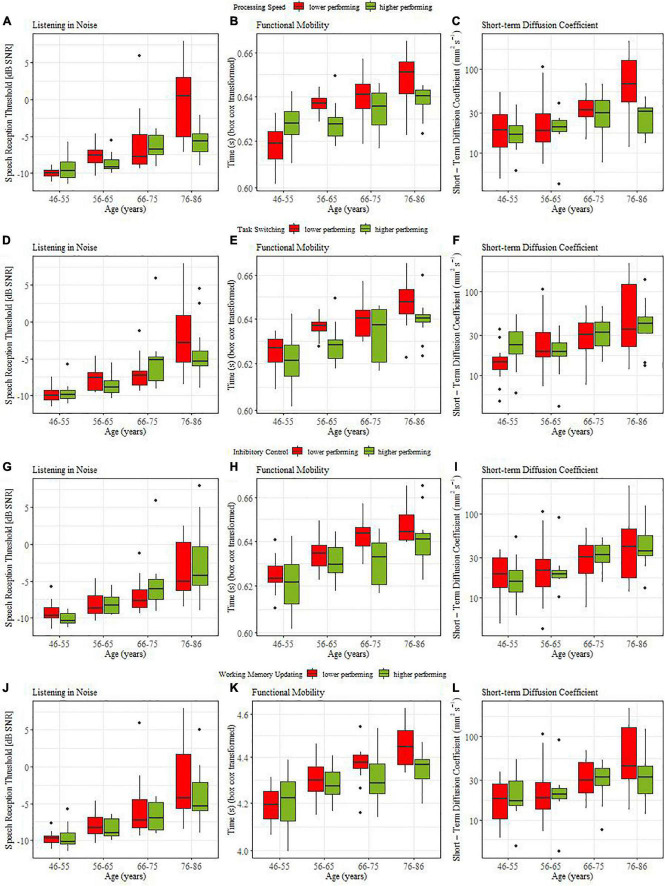
Coupling between sensorimotor and cognitive functions for lower (red) and higher (green) performing participants.

From top to bottom, Figures 3A,D,G,J illustrate the coupling between listening in noise thresholds for individuals with high (green bars) versus low (red bars) cognitive ability (assessed by processing speed, task switching, inhibitory control, and working memory updating, respectively). None of the median splits based on cognitive abilities induced a main effect. However, when processing speed was used to distinguish high and low cognitive performers, we obtained a significant interaction with the contrast comparing young vs. oldest old (β = −0.004, SE = 0.14, *t* = 2.71, *p* = = 0.008). *Post hoc* analysis showed that only the oldest age group (76-86 yr) yielded a reliable coupling in that older adults with slower processing speed also required reliably higher thresholds during listening in noise (ΔM = 0.07, *t*(22) = 3.45, *p* = 0.002).

Figures 3B,E,H,K illustrate the coupling between functional mobility (TUG) and the four cognitive variables. For all cognitive abilities we obtained main effects of median split: processing speed (β = −0.002, SE = 0.009, *t* = 2.30, *p* = 0.032), for task switching (β = −0.003, SE = 0.001, *t* = 3.06, *p* = 0.004), for inhibitory control (β = −0.003, SE = 0.001, *t* = 3.255, *p* = 0.002 0.05), and for working memory updating (β = −0.002, SE = 0.002, *t* = 2.55, *p* = 0.026). For processing speed and working memory updating significant interaction effects with age group contrasts were obtained. For processing speed this involved middle-aged versus old (β = −0.008, SE = 0.004, *t* = 2.17, *p* = 0.021), and young middle-aged (46-55yr.) vs. older middle-aged (56-65 yr) individuals (β = −0.008, SE = 0.002, *t* = 2.96, *p* = 0.002). *Post hoc* t-tests showed that in the 56-65yr (ΔM = 0.008, *t*(18.5) = 2.85, *p* = 0.012) and 76-86yr old groups (ΔM = 0.01, *t*(17) = 2.62, *p* = 0.018) individuals with higher processing speed also showed better functional mobility. For working memory updating the interaction (β = −0.009, SE = 0.004, *t* = 2.26, *p* = 0.026) indicated that in middle-aged individuals, cognition was not coupled to functional mobility while older adults with better working memory had higher functional mobility (ΔM = 0.01, *t*(46) = 2.96, *p* = 0.017). In sum, we found strong evidence for a coupling between cognitive abilities and functional mobility. This coupling was similar across age groups for switching and inhibition while coupling increased with age when processing speed or working memory updating were considered.

Figures 3C,F,I,L illustrate the coupling between postural control (short-term diffusion coefficient) and cognitive ability. Only processing speed yielded significant coupling effects, namely interactions of median split with the middle-aged versus old contrast (β = −0.26, SE = 0.11, *t* = 2.34, *p* = 0.033), and the young-old (66-75yr) versus old-old (76-86yr) contrast, (β = −0.16, SE = 0.07, *t* = 2.34, *p* = 0.034 0.05). *Post hoc* t-tests confirmed that older adults with faster processing speed had better postural control (ΔM = 0.23, *t*(41.2) = 2.7, *p* = 0.008) and that this coupling relation was pronounced for the comparison within older age groups (ΔM = 0.40, *t*(17.1) = 3.25, *p* = 0.005).

## Discussion

The present study explored age effects and the coupling of sensorimotor and cognitive functions during middle- and late adulthood in individuals from four age groups with no indication of even mild cognitive decline. In a first step, we aimed to establish negative age-related differences for cognitive as well as sensorimotor functions, as could be expected based on extensive earlier research. Naturally, such demonstration is a prerequisite to exploring the coupling of sensorimotor and cognitive functions and their age-related intensification. Following the different theoretical accounts (common cause, cascade, compensation), we expected this coupling to emerge during late middle adulthood and to increase in the older age groups.

Except for tactile sensitivity, all measures yielded substantial age effects consistent with those reported in the literature. The median value of the SRT in the 46-55 group, −10 dB SNR, corresponds to the normative value for good hearing in young and middle-aged adults (Jansen et al., 2013; Vercammen et al., 2018a). The decline in speech in noise by about 0.15 dB per annum is comparable to the 0.18 dB SNR reported by Pronk et al. (2013) for persons between 57 and 93 years of age using a similar digits-in-noise task. Like in their study, the rate accelerated with age in our sample (0.16, 0.19, 0.32 dB SNR between the four age cohorts), caused by alterations in peripheral auditory, central auditory and cognitive changes.

For postural control, we found relative age-graded stability until late middle adulthood with substantial performance decrements in later decades of life. This is in line with normative data from Abrahamová and Hlavacka (2008) and Goble and Baweja (2018), who demonstrated strong age effects from the seventh decade onward. In our study, the short-term diffusion coefficient was the only variable among the SDA parameters sensitive to the balance system’s age-related differences. This suggests that open-loop control processes are most affected by this age-related deterioration. Surprisingly, no differences were found for short-term diffusion coefficients when we compared the two oldest age groups. One potential explanation for this is the relatively low complexity of the postural control task; studies have indicated that age effects in postural control become more pronounced with increasing task complexity (Boisgontier et al., 2013; Carr et al., 2020). In line with Laughton et al. (2003) and Norris et al. (2005), no age-related differences were observed for the long-term diffusion coefficient. The critical time interval also did not show any age-related differences. This is in contrast with earlier studies that found a substantial increase in the critical time interval with increasing age (Collins and De Luca, 1995; Norris et al., 2005). As these studies compared young to older adults and averaged the mean squared displacement of 10 trials to calculate their SDA variables, methodological discrepancies are most likely responsible for these differences.

For functional mobility, we observed, on average, an increase in TUG-times similar to the 0.6-0.8 second increase per decade reported by Vereeck et al. (2008). Our assessment of tactile sensitivity only revealed reliable differences between young and older middle-age and age-graded stability in later phases. Most studies found an accelerated decrease in tactile perception with advanced age. As Berquin et al. (2010) observed significant effects of age only in the upper limbs but not in the lower limbs, we believe that our method, which measures at the feet, may be suboptimal for capturing changes in the older cohorts.

Processing speed and measures of cognitive control also showed robust age effects except for specific switch costs. It is well known that older adults need more time to process information than younger ones (Salthouse, 1996, 2009), that interference control changes with increasing age using the Stroop task (Gajewski et al., 2020), and that working memory updating is subject to age (De Beni and Palladino, 2004). Absence of age effects in specific task-switching costs was also reported by Verhaeghen and Cerella (2002) when reviewing the results of a series of meta-analyses examining age-related differences in selective attention (e.g., Stroop task) and divided attention (task switching).

We took two different approaches to explore sensorimotor coupling and its age-related intensification. The first approach was inspired by earlier common cause research investigating how much age-related variance (ARV) in cognitive functions could be explained by processing speed or sensorimotor functions. In line with the results presented by Lindenberger et al. (1993), we found that most of the age-related variance in cognitive control measures was mediated by processing speed. Mediator effects were generally much lower for working memory updating. Functional mobility turned out to be almost as successful as a mediator of ARV, in line with the findings presented by Lindenberger and Baltes (1994). Different from our expectations, the other two sensorimotor functions listening in noise and short-term diffusion in postural control, were poor mediators of ARV in cognitive functions.

For our second approach to coupling, we split individuals in the four age groups into high- and low-ability subgroups based on their performances in four markers of domain-general cognitive functioning. Subsequently, we asked whether high vs. low cognitive ability corresponded to better vs. poor performance levels in three sensorimotor functions. Like before, we found the strongest evidence for sensorimotor-cognition coupling when processing speed was used to identify subgroups with high vs. low cognitive abilities. Processing speed accounted for individual differences in all three sensorimotor functions, and this coupling increased with advancing ages for listening in noise, functional mobility, as well as postural control. For markers of cognitive control (inhibition, switching, working memory updating), the evidence was mixed. For all three markers, we demonstrated significant coupling with individual differences in functional mobility and working memory updating also showed increased coupling strength with age, as expected. At the same time, no indication of coupling was evident for cognitive control functions and listening in noise or postural control.

In sum, except for tactile sensitivity, we found substantial age-related differences in the sensorimotor and cognitive tasks, which was perfectly in line with previous studies and sufficient grounds for our investigation of sensorimotor-cognitive coupling and its age-related intensification. Different from our expectations and earlier studies we found that only processing speed and functional mobility reliably showed coupling and age-related increases thereof in combination with different cognitive or sensorimotor variables. When we extended the functions considered to cognitive control on the one hand and posture or listening in noise on the other, evidence for coupling was weak. The bottom line is that individuals with poor cognitive control do not necessarily have poor listening-in-noise skills or poor postural control.

### Limitations and associations

Our study was explorative by its correlational and cross-sectional design, as is the bulk of the evidence accumulated to support cascade models, common-cause or cognitive compensation hypotheses. For some time, researchers have recognized that a solid evaluation and comparison of the different accounts require longitudinal data and sophisticated approaches (Ghisletta and Lindenberger, 2005; Kiely and Anstey, 2015). Nevertheless, even more sophisticated approaches yield moderate correlations between sensory and cognitive declines (Lindenberger and Ghisletta, 2009.

Key differences with earlier studies relate to our choices for sensorimotor and cognitive functions and how we assessed performance. While most studies compared young and old individuals, we narrowed the age range to periods where functional decline has been demonstrated to accelerate and during which the coupling of sensorimotor and cognitive functions is assumed to become stronger. In our study, the coupling in middle-aged is not very pronounced, perhaps because only 7% of our participants wore hearing aids (compared to 16.7% in the study by Lindenberger and Baltes, 1994). Analyses of 165.000 persons between 49*-*69 years showed that 10.7% of adults had significant hearing impairment based on a similar digits-in-noise task (Dawes et al., 2014). In our sample the prevalence of HI is 4% for 46-55 years, 21% for 56-65 years, 46% for 66-75 years and 83% for 76-86 years based on a cut-off of –7 dB SNR. This cut-off is lower than the reference value at –8.6 dB SNR for middle-aged persons (Vercammen et al., 2018a), indicating that these persons are likely to have HI.

Our mixed results as far as coupling between different cognitive and sensorimotor abilities go is not an exception in the literature. For example, Dryden et al. (2017) reported variable associations between cognition and speech in noise understanding. Their systematic study showed that the overall association between cognitive performance and speech understanding in noise was in the order of *r* = 0.31. More recently, Danielsson et al. (2019) showed that the association between age, auditory function, and cognition looked different depending on the type of variable used to represent auditory function and cognition. In our study listening in noise was assessed with a speech-weighted noise task which is cognitively less demanding than an informational masker (e.g., Goossens et al., 2017). In a similar vein, no significant correlations were observed between amplitude modulation detection thresholds for diotic tones and cognitive abilities (Füllgrabe et al., 2015), while strong correlations were observed between spatial audition and performance on the trail-making task in older persons with HI (Strelcyk et al., 2019), presumably because spatial cues are coded centrally. A modality-general spatial processing deficit and/or individual differences in global processing speed could lie at the basis of this relationship. Previously, significant correlations were also observed between the temporal fine structure of the signal and cognitive factors (Rönnberg et al., 2016; Ellis and Rönnberg, 2022).

### Implications for hearing rehabilitation

Although more than 70% of listeners with self-reported hearing problems mention having consulted a medical professional about their hearing health (Laureyns et al., 2016), hearing aid uptake in this group ranges from 20-40% only (Abrams and Kihm, 2015; Hougaard et al., 2016; Laureyns et al., 2016). This is unfortunate as hearing aid use is associated with better cognition, independently of social isolation and depression (Dawes et al., 2015). Hearing aids may improve cognitive performance, presumably because of improvement in audibility or associated increases in self-efficacy. Similarly, improvements in working memory and processing speed were reported for persons over 70 years with bilateral hearing impairment following unilateral cochlear implantation (Knopke et al., 2021). Besides technological intervention, auditory training involving cognitive processes may also improve working memory and other cognitive factors (Ferguson and Henshaw, 2015). As the sensorimotor-cognitive coupling was strongest in the older age groups it may be that training processing speed improves listening skills and indirectly other sensorimotor skills like keeping posture and walking.

## Conclusion

We demonstrated robust age effects for cognitive and sensorimotor functions emerging in middle adulthood and accelerating in late adulthood. Processing speed and functional mobility reflected sensorimotor-cognition coupling and its age-related intensification. However, this was not true for other domain-general cognitive abilities and sensorimotor functions. A major implication is that domain-specific factors must also play a major role in cognitive and sensorimotor aging. While this might complicate theorizing, it portrays an optimistic perspective in our view in that it “does NOT go altogether when it goes” (Rabbitt, 1993). Further research is needed to establish the relationship between the cognitive constructs and sensorimotor functioning in aging individuals in order to develop targeted interventions for persons with HI.

## Data availability statement

The raw data supporting the conclusions of this article will be made available upon request.

## Ethics statement

The studies involving human participants were reviewed and approved by Medical Ethical Committee of UZ Leuven/KU Leuven. The patients/participants provided their written informed consent to participate in this study.

## Author contributions

MV and NV prepared the materials, collected the data, and preprocessed the data. MV performed the data analysis. All authors have contributed to the study conception and design, each wrote parts of the manuscript, and provided critical revision and feedback.

## Footnotes

1
www.who.int

2
https://www.nice.org.uk/guidance/cg161

## References

[B1] Abrahamová D. Hlavacka F. (2008). Age-related changes of human balance during quiet stance. *Physiol. Res.* 57 957–964. 10.33549/physiolres.931238 18052683

[B2] Abrams H. B. Kihm J. (2015). An introduction to marketrak IX: A new baseline for the hearing aid market. *Hear. Rev.* 22:16.

[B3] Anderson S. Bieber R. Schloss A. (2021). Peripheral deficits and phase-locking declines in aging adults. *Hear. Res.* 403:108188. 10.1016/j.heares.2021.108188 33581668 PMC7980782

[B4] Anderson S. Parbery-Clark A. White-Schwoch T. Kraus N. (2012). Aging affects neural precision of speech encoding. *J. Neurosci.* 32 14156–14164. 10.1523/JNEUROSCI.2176-12.2012 23055485 PMC3488287

[B5] Baltes P. B. Staudinger U. M. Lindenberger U. (1999). Lifespan psychology: Theory and application to intellectual functioning. *Annu. Rev. Psychol.* 50 471–507. 10.1146/annurev.psych.50.1.471 15012462

[B6] Belmin J. Pariel-Madjlessi S. Surun P. Bentot C. Feteanu D. Lefebvre des Noettes V. (2007). The cognitive disorders examination (Codex) is a reliable 3-minute test for detection of dementia in the elderly (validation study on 323 subjects). *Presse Med.* 36 1183–1190. 10.1016/j.lpm.2007.03.016 17433613

[B7] Berquin A. D. Lijesevic V. Blond S. Plaghki L. (2010). An adaptive procedure for routine measurement of light-touch sensitivity threshold. *Muscle Nerve.* 42 328–338. 10.1002/mus.21689 20665509

[B8] Berryman N. Bherer L. Nadeau S. Lauzière S. Lehr L. Bobeuf F. (2013). Executive functions, physical fitness and mobility in well-functioning older adults. *Exp. Gerontol.* 48 1402–1409. 10.1016/j.exger.2013.08.017 24012563

[B9] Boisgontier M. P. Beets I. A. Duysens J. Nieuwboer A. Krampe R. T. Swinnen S. P. (2013). Age-related differences in attentional cost associated with postural dual tasks: Increased recruitment of generic cognitive resources in older adults. *Neurosci. Biobehav. Rev.* 37 1824–1837. 10.1016/j.neubiorev.2013.07.014 23911924

[B10] Bouça-Machado R. Duarte G. S. Patriarca M. Castro Caldas A. Alarcão J. Fernandes R. M. (2020). Measurement instruments to assess functional mobility in Parkinson’s disease: A systematic review. *Mov. Disord. Clin. Pract.* 7 129–139. 10.1002/mdc3.12874 32071930 PMC7011644

[B11] Brodoehl S. Klingner C. Stieglitz K. Witte O. W. (2013). Age-related changes in the somatosensory processing of tactile stimulation–an fMRI study. *Behav. Brain Res.* 238 259–264. 10.1016/j.bbr.2012.10.038 23123141

[B12] Butler A. A. Menant J. C. Tiedemann A. C. Lord S. R. (2009). Age and gender differences in seven tests of functional mobility. *J. Neuroeng. Rehabil.* 6:31. 10.1186/1743-0003-6-31 19642991 PMC2741473

[B13] Cabeza R. Grady C. L. Nyberg L. McIntosh A. R. Tulving E. Kapur S. (1997). Age-related differences in neural activity during memory encoding and retrieval: A positron emission tomography study. *J. Neurosci.* 17 391–400. 8987764 10.1523/JNEUROSCI.17-01-00391.1997 PMC6793692

[B14] Carr S. Pichora-Fuller M. K. Li K. Z. H. Campos J. L. (2020). Effects of age on listening and postural control during realistic multi-tasking conditions. *Hum. Mov. Sci.* 73:102664. 10.1016/j.humov.2020.102664 32768861

[B15] Cerella J. (1985). Information processing rates in the elderly. *Psychol. Bull.* 98 67–83. 4034819

[B16] Collins J. J. De Luca C. J. (1993). Open-loop and closed-loop control of posture: A random-walk analysis of center-of-pressure trajectories. *Exp. Brain Res.* 95 308–318. 10.1007/BF00229788 8224055

[B17] Collins J. J. De Luca C. J. (1995). Age-related changes in open-loop and closed-loop postural control mechanisms. *Exp. Brain Res.* 104 480–492. 10.1007/BF00231982 7589299

[B18] Cooper J. Siegfried K. Ahmed A. A. (2014). *BrainBLoX: Brain and biomechanics lab in a box software (Version 1.0) [Software].* Available online at: http://www.colorado.edu/neuromechanics/research/wii-balance-board-project

[B19] D’Esposito M. Postle B. R. (2015). The cognitive neuroscience of working memory. *Annu. Rev. Psychol.* 66 115–142. 25251486 10.1146/annurev-psych-010814-015031 PMC4374359

[B20] Danielsson H. Humes L. E. Rönnberg J. (2019). Different associations between auditory function and cognition depending on type of auditory function and type of cognition. *Ear Hear.* 40 1210–1219. 10.1097/AUD.0000000000000700 30807540 PMC6706331

[B21] Dawes P. Emsley R. Cruickshanks K. J. Moore D. R. Fortnum H. Edmondson-Jones M. (2015). Hearing loss and cognition: The role of hearing AIDS, social isolation and depression. *PLoS One* 10:e0119616. 10.1371/journal.pone.0119616 25760329 PMC4356542

[B22] Dawes P. Fortnum H. Moore D. R. Emsley R. Norman P. Cruickshanks K. (2014). Hearing in middle age: A population snapshot of 40- to 69-year olds in the United Kingdom. *Ear Hear.* 35 e44–e51. 10.1097/AUD.0000000000000010 24518430 PMC4264521

[B23] De Beni R. Palladino P. (2004). Decline in working memory updating through ageing: Intrusion error analyses. *Memory* 12 75–89. 10.1080/0965821024400056 15098622

[B24] Denys S. Hofmann M. van Wieringen A. Wouters J. (2019). Improving the efficiency of the digit triplet test using digit scoring with variable adaptive step sizes. *Int. J. Audiol.* 58 670–677. 10.1080/14992027.2019.1622042 31187664

[B25] Desjardins-Crépeau L. Berryman N. Vu T. T. Villalpando J. M. Kergoat M. J. Li K. Z. (2014). Physical functioning is associated with processing speed and executive functions in community-dwelling older adults. *J. Gerontol. B Psychol. Sci. Soc. Sci.* 69 837–844. 10.1093/geronb/gbu036 24829304

[B26] Dryden A. Allen H. A. Henshaw H. Heinrich A. (2017). The association between cognitive performance and speech-in-noise perception for adult listeners: A Systematic literature review and meta-analysis. *Trends Hear.* 21 1–21. 10.1177/2331216517744675 29237334 PMC5734454

[B27] Dyck P. J. O’Brien P. C. Kosanke J. L. Gillen D. A. Karnes J. L. (1993). A 4, 2, and 1 stepping algorithm for quick and accurate estimation of cutaneous sensation threshold. *Neurology* 43 1508–1512. 10.1212/wnl.43.8.1508 8351003

[B28] Ellis R. J. Rönnberg J. (2022). Temporal fine structure: Associations with cognition and speech-in-noise recognition in adults with normal hearing or hearing impairment. *Int. J. Audiol.* 61 778–786. 10.1080/14992027.2021.1948119 34292115

[B29] England D. Ruddy K. L. Dakin C. J. Schwartz S. E. Butler B. Bolton D. A. E. (2021). Relationship between speed of response inhibition and ability to suppress a step in midlife and older adults. *Brain Sci.* 11:643. 10.3390/brainsci11050643 34063458 PMC8156272

[B30] Ferguson M. A. Henshaw H. (2015). Auditory training can improve working memory, attention, and communication in adverse conditions for adults with hearing loss. *Front. Psychol.* 28:556. 10.3389/fpsyg.2015.00556 26074826 PMC4447061

[B31] Finley J. M. Dhaher Y. Y. Perreault E. J. (2012). Contributions of feed-forward and feedback strategies at the human ankle during control of unstable loads. *Exp. Brain Res.* 217 53–66. 10.1007/s00221-011-2972-9 22169978 PMC3593720

[B32] Fox J. Weisberg S. (2019). *An R companion to applied regression, Third edition.* Thousand Oaks CA: Sage.

[B33] Fuller G. F. (2000). Falls in the elderly. *Am. Fam. Physician* 61 2173–2174. 10779256

[B34] Füllgrabe C. Moore B. C. Stone M. A. (2015). Age-group differences in speech identification despite matched audiometrically normal hearing: Contributions from auditory temporal processing and cognition. *Front. Aging Neurosci.* 13:347. 10.3389/fnagi.2014.00347 25628563 PMC4292733

[B35] Gajewski P. D. Falkenstein M. Thönes S. Wascher E. (2020). Stroop task performance across the lifespan: High cognitive reserve in older age is associated with enhanced proactive and reactive interference control. *Neuroimage* 207:116430. 10.1016/j.neuroimage.2019.116430 31805383

[B36] Gajewski P. D. Hanisch E. Falkenstein M. Thönes S. Wascher E. (2018). What does the n-back task measure as we get older? Relations between working-memory measures and other cognitive functions across the lifespan. *Front. Psychol.* 26:2208. 10.3389/fpsyg.2018.02208 30534095 PMC6275471

[B37] Gao X. Wehr M. (2015). A coding transformation for temporally structured sounds within auditory cortical neurons. *Neuron* 86 292–303. 10.1016/j.neuron.2015.03.004 25819614 PMC4393373

[B38] Gerver C. R. Neely K. A. Kurkela K. A. Diaz M. T. Goodman J. T. Blouch S. (2020). Shared neural recruitment across working memory and motor control tasks as a function of task difficulty and age. *Aging Neuropsychol. Cogn.* 27 864–879. 10.1080/13825585.2019.1700898 31877068 PMC7316585

[B39] Ghisletta P. Lindenberger U. (2005). Exploring structural dynamics within and between sensory and intellectual functioning in old and very old age: Longitudinal evidence from the Berlin aging study. *Intelligence* 33 555–587. 10.1016/j.intell.2005.07.002

[B40] Goble D. J. Baweja H. S. (2018). Postural sway normative data across the adult lifespan: Results from 6280 individuals on the balance tracking system balance test. *Geriatr. Gerontol. Int.* 18 1225–1229. 10.1111/ggi.13452 29897159

[B41] Goble D. J. Coxon J. P. Van Impe A. De Vos J. Wenderoth N. Swinnen S. P. (2010). The neural control of bimanual movements in the elderly: Brain regions exhibiting age-related increases in activity, frequency-induced neural modulation, and task-specific compensatory recruitment. *Hum. Brain Mapp.* 31 1281–1295. 10.1002/hbm.20943 20082331 PMC6871108

[B42] Goossens T. Vercammen C. Wouters J. van Wieringe N. A. (2018a). Neural envelope encoding predicts speech perception performance for normal-hearing and hearing-impaired adults. *Hear. Res.* 370 189–200. 10.1016/j.heares.2018.07.012 30131201

[B43] Goossens T. Vercammen C. Wouters J. van Wieringen A. (2017). Masked speech perception across the adult lifespan: Impact of age and hearing impairment. *Hear. Res.* 344 109–124. 27845259 10.1016/j.heares.2016.11.004

[B44] Goossens T. Vercammen C. Wouters J. van Wieringen A. (2018b). The association between hearing impairment and neural envelope encoding at different ages. *Neurobiol. Aging* 11 202–212. 10.1016/j.neurobiolaging.2018.10.008 30472387

[B45] Gordon-Salant S. Cole S. S. (2016). Effects of age and working memory capacity on speech recognition performance in noise among listeners with normal hearing. *Ear Hear.* 37 593–602. 10.1097/AUD.0000000000000316 27232071

[B46] Gordon-Salant S. Fitzgibbons P. J. (1999). Profile of auditory temporal processing in older listeners. *J. Speech Lang. Hear. Res.* 42 300–311. 10.1044/jslhr.4202.300 10229448

[B47] Goy H. Pelletier M. Coletta M. Pichora-Fuller M. K. (2013). The effects of semantic context and the type and amount of acoustic distortion on lexical decision by younger and older adults. *J. Speech Lang. Hear. Res.* 56 1715–1732. 10.1044/1092-4388(2013/12-0053) 23882006

[B48] Guralnik J. M. Simonsick E. M. Ferrucci L. Glynn R. J. Berkman L. F. Blazer D. G. (1994). A short physical performance battery assessing lower extremity function: Association with self-reported disability and prediction of mortality and nursing home admission. *J. Gerontol.* 49 M85–M94. 10.1093/geronj/49.2.m85 8126356

[B49] Helfer K. S. Freyman R. L. (2008). Aging and speech-on-speech masking. *Ear Hear.* 29 87–98. 10.1097/AUD.0b013e31815d638b 18091104 PMC2987598

[B50] Heuninckx S. Wenderoth N. Swinnen S. P. (2008). Systems neuroplasticity in the aging brain: Recruiting additional neural resources for successful motor performance in elderly persons. *J. Neurosci.* 28 91–99. 10.1523/jneurosci.3300-07.2008 18171926 PMC6671150

[B51] Heuninckx S. Wenderoth N. Swinnen S. P. (2010). Age-related reduction in the differential pathways involved in internal and external movement generation. *Neurobiol. Aging* 31 301–314. 10.1016/j.neurobiolaging.2008.03.021 18472185

[B52] Hougaard S. Ruf S. Egger C. Abrams H. (2016). Hearing aids improve hearing–and a lot more. *Hear. Rev.* 23:14. 10.1016/s0140-6736(02)08433-7 11988245

[B53] Humes L. E. Young L. A. (2016). Sensory–cognitive interactions in older adults. *Ear Hear.* 37 52S–61S. 27355770 10.1097/AUD.0000000000000303 PMC4930008

[B54] Ibrahim A. Singh D. K. A. Shahar S. (2017). ’Timed Up and Go’ test: Age, gender and cognitive impairment stratified normative values of older adults. *PLoS One* 12:e0185641–e0185641. 10.1371/journal.pone.0185641 28972994 PMC5626462

[B55] Jaeger J. (2018). Digit symbol substitution test: The case for sensitivity over specificity in neuropsychological testing. *J. Clin. Psychopharmacol.* 38 513–519. 10.1097/JCP.0000000000000941 30124583 PMC6291255

[B56] Janse E. (2012). A non-auditory measure of interference predicts distraction by competing speech in older adults. *Aging Neuropsychol. Cogn.* 19 741–758. 10.1080/13825585.2011.652590 22293017

[B57] Jansen S. Luts H. Dejonckere P. van Wieringen A. Wouters J. (2013). Efficient hearing screening in noise-exposed listeners using the digit triplet test. *Ear Hear.* 34 773–778. 10.1097/AUD.0b013e318297920b 23782715

[B58] Jayakody D. M. P. Friedland P. L. Martins R. N. Sohrabi H. R. (2018). Impact of aging on the auditory system and related cognitive functions: A narrative review. *Front. Neurosci.* 5:125. 10.3389/fnins.2018.00125 29556173 PMC5844959

[B59] Johannsen L. Van Humbeeck N. Krampe R. (2022). “Multitasking during continuous task demands: The cognitive costs of concurrent sensorimotor activities,” in *Handbook of human multitasking*, eds Kiesel A. Johannsen L. Koch I. Müller H. (Cham: Springer Nature), 37–81. 10.1007/978-3-031-04760-2_2

[B60] Khant N. Dani V. B. Patel P. Rathod R. (2018). Establishing the reference value for “timed up-and-go” test in healthy adults of Gujarat, India. *J. Educ. Health Promot.* 7 62–62. 10.4103/jehp.jehp_12_18 29922691 PMC5963216

[B61] Kidd G. Jr. Arbogast T. L. Mason C. R. Gallun F. J. (2005). The advantage of knowing where to listen. *J. Acoust. Soc. Am.* 118 3804–3815. 16419825 10.1121/1.2109187

[B62] Kiely K. M. Anstey K. J. (2015). “Common cause theory in aging,” in *Encyclopedia of geropsychology*, ed. Pachana N. A. (Singapore: Springer), 10.1007/978-981-287-080-3_118-1

[B63] Killane I. Donoghue O. A. Savva G. M. Cronin H. Kenny R. A. Reilly R. B. (2014). Relative association of processing speed, short-term memory and sustained attention with task on gait speed: A study of community-dwelling people 50 years and older. *J. Gerontol. A Biol. Sci. Med. Sci.* 69 1407–1414. 10.1093/gerona/glu140 25182598

[B64] Knight S. Heinrich A. (2017). Different measures of auditory and visual stroop interference and their relationship to speech intelligibility in noise. *Front. Psychol.* 8:230. 10.3389/fpsyg.2017.00230 28367129 PMC5355492

[B65] Knopke S. Schubert A. Häussler S. M. Gräbel S. Szczepek A. J. Olze H. (2021). Improvement of working memory and processing speed in patients over 70 with bilateral hearing impairment following unilateral cochlear implantation. *J. Clin. Med.* 10:3421. 10.3390/jcm10153421 34362204 PMC8347702

[B66] Koerner T. K. Muralimanohar R. K. Gallun F. J. Billings C. J. (2020). Age-related deficits in electrophysiological and behavioral measures of binaural temporal processing. *Front. Neurosci.* 14:578566. 10.3389/fnins.2020.578566 33192263 PMC7654338

[B67] Krampe R. T. (2002). Aging, expertise and fine motor movement. *Neurosci. Biobehav. Rev.* 26 769–776. 10.1016/s0149-7634(02)00064-7 12470688

[B68] Kray J. Lindenberger U. (2000). Adult age differences in task switching. *Psychol. Aging* 15 126–147. 10.1037//0882-7974.15.1.126 10755295

[B69] Kujawa S. G. Liberman M. C. (2015). Synaptopathy in the noise-exposed and aging cochlea: Primary neural degeneration in acquired sensorineural hearing loss. *Hear. Res.* 2015 191–199. 10.1016/j.heares.2015.02.009 25769437 PMC4567542

[B70] Kwag E. Zijlstra W. (2022). Balance tasks requiring inhibitory control; a scoping review of studies in older adults. *Gait. Posture* 93 126–134. 10.1016/j.gaitpost.2022.01.025 35139472

[B71] Lacour M. Bernard-Demanze L. Dumitrescu M. (2008). Posture control, aging, and attention resources: Models and posture-analysis methods. *Neurophysiol. Clin.* 38 411–421. 10.1016/j.neucli.2008.09.005 19026961

[B72] Laughton C. A. Slavin M. Katdare K. Nolan L. Bean J. F. Kerrigan D. C. (2003). Aging, muscle activity, and balance control: Physiologic changes associated with balance impairment. *Gait Posture* 18 101–108. 10.1016/s0966-6362(02)00200-x 14654213

[B73] Laureyns M. Best L. Bisgaard N. Hougaard S. (2016). *Getting our numbers right on hearing loss – Hearing care and hearing aid use in Europe. Joint AEA, EFHOH and EHIMA report*. Available online at: www.ehima.com

[B74] Lawo V. Koch I. (2014). Examining age-related differences in auditory attention control using a task-switching procedure. *J. Gerontol. B Psychol. Sci. Soc. Sci.* 69 237–244. 10.1093/geronb/gbs107 23197343

[B75] Li K. Z. Lindenberger U. (2002). Relations between aging sensory/sensorimotor and cognitive functions. *Neurosci. Biobehav. Rev.* 26 777–783. 10.1016/s0149-7634(02)00073-8 12470689

[B76] Li K. Z. Lindenberger U. Freund A. M. Baltes P. B. (2001). Walking while memorizing: Age-related differences in compensatory behavior. *Psychol. Sci.* 12 230–237. 10.1111/1467-9280.00341 11437306

[B77] Lindenberger U. Baltes P. B. (1994). Sensory functioning and intelligence in old age: A strong connection. *Psychol. Aging* 9 339–355. 10.1037//0882-7974.9.3.339 7999320

[B78] Lindenberger U. Ghisletta P. (2009). Cognitive and sensory declines in old age: Gauging the evidence for a common cause. *Psychol. Aging* 24 1–16. 10.1037/a0014986 19290733

[B79] Lindenberger U. Marsiske M. Baltes P. B. (2000). Memorizing while walking: Increase in dual-task costs from young adulthood to old age. *Psychol. Aging* 15 417–436. 10.1037//0882-7974.15.3.417 11014706

[B80] Lindenberger U. Mayr U. Kliegl R. (1993). Speed and intelligence in old age. *Psychol. Aging* 8 207–220. 10.1037//0882-7974.8.2.207 8323725

[B81] Livingston G. Huntley J. Sommerlad A. Ames D. Ballard C. Banerjee S. (2020). Dementia prevention, intervention, and care: 2020 report of the lancet commission. *Lancet* 396 413–446. 32738937 10.1016/S0140-6736(20)30367-6 PMC7392084

[B82] Lowry K. A. Brach J. S. Nebes R. D. Studenski S. A. VanSwearingen J. M. (2012). Contributions of cognitive function to straight- and curved-path walking in older adults. *Arch. Phys. Med. Rehabil.* 93 802–807. 10.1016/j.apmr.2011.12.007 22541307 PMC4878139

[B83] MacLeod C. M. (1991). Half a century of research on the stroop effect: An integrative review. *Psychol. Bull.* 109 163–203. 10.1037/0033-2909.109.2.163 2034749

[B84] Mathôt S. Schreij D. Theeuwes J. (2012). OpenSesame: An open-source, graphical experiment builder for the social sciences. *Behav. Res. Methods* 44 314–324. 10.3758/s13428-011-0168-7 22083660 PMC3356517

[B85] Michalska J. Kamieniarz A. Sobota G. Stania M. Juras G. Słomka K. J. (2021). Age-related changes in postural control in older women: Transitional tasks in step initiation. *BMC Geriatr.* 21:17. 10.1186/s12877-020-01985-y 33407197 PMC7789726

[B86] Mirelman A. Herman T. Brozgol M. Dorfman M. Sprecher E. Schweiger A. (2012). Executive function and falls in older adults: New findings from a five-year prospective study link fall risk to cognition. *PLoS One* 2012:e40297. 10.1371/journal.pone.0040297 22768271 PMC3386974

[B87] Miyake A. Friedman N. P. (2012). The nature and organization of individual differences in executive functions: Four general conclusions. *Curr. Dir. Psychol. Sci.* 21 8–14. 10.1177/0963721411429458 22773897 PMC3388901

[B88] Miyake A. Friedman N. P. Emerson M. J. Witzki A. H. Howerter A. Wager T. D. (2000). The unity and diversity of executive functions and their contributions to complex ‘Frontal Lobe’ tasks: A latent variable analysis. *Cogn. Psychol.* 41 49–100. 10.1006/cogp.1999.0734 10945922

[B89] Moore B. C. Vickers D. A. Mehta A. (2012). The effects of age on temporal fine structure sensitivity in monaural and binaural conditions. *Int. J. Audiol.* 51 715–721. 10.3109/14992027.2012.690079 22998412

[B90] Myerson J. Hale S. Wagstaff D. Poon L. W. Smith G. A. (1990). The information-loss model: A mathematical theory of age-related cognitive slowing. *Psychol. Rev.* 97 475–487. 10.1037/0033-295x.97.4.475 2247538

[B91] Noble W. Jensen N. S. Naylor G. Bhullar N. Akeroyd M. A. (2013). A short form of the speech, spatial and qualities of hearing scale suitable for clinical use: The SSQ12. *Int. J. Audiol.* 52 409–412. 10.3109/14992027.2013.781278 23651462 PMC3864780

[B92] Norris J. A. Marsh A. P. Smith I. J. Kohut R. I. Miller M. E. (2005). Ability of static and statistical mechanics posturographic measures to distinguish between age and fall risk. *J. Biomech.* 38 1263–1272. 10.1016/j.jbiomech.2004.06.014 15863111

[B93] Oberem J. Koch I. Fels J. (2017). Intentional switching in auditory selective attention: Exploring age-related effects in a spatial setup requiring speech perception. *Acta Psychol. (Amst)* 177 36–43. 10.1016/j.actpsy.2017.04.008 28456098

[B94] Parthasarathy A. Kujawa S. G. (2018). Synaptopathy in the aging cochlea: Characterizing early-neural deficits in auditory temporal envelope processing. *J. Neurosci.* 38 7108–7119. 10.1523/JNEUROSCI.3240-17.2018 29976623 PMC6596096

[B95] Perrone-Bertolotti M. Tassin M. Meunier F. (2017). Speech-in-speech perception and executive function involvement. *PLoS One* 12:e0180084. 10.1371/journal.pone.0180084 28708830 PMC5510830

[B96] Perry S. D. (2006). Evaluation of age-related plantar-surface insensitivity and onset age of advanced insensitivity in older adults using vibratory and touch sensation tests. *Neurosci. Lett.* 392 62–67. 10.1016/j.neulet.2005.08.060 16183200

[B97] Plomp R. (1978). Auditory handicap of hearing impairment and the limited benefit of hearing aids. *J. Acoust. Soc. Am.* 63 533–549. 10.1121/1.381753 670550

[B98] Podsiadlo D. Richardson S. (1991). The timed “Up & Go”: A test of basic functional mobility for frail elderly persons. *J Am. Geriatr. Soc.* 39 142–148. 10.1111/j.1532-5415.1991.tb01616.x 1991946

[B99] Presacco A. Simon J. Z. Anderson S. (2016). Evidence of degraded representation of speech in noise, in the aging midbrain and cortex. *J. Neurophysiol.* 116 2346–2355. 10.1152/jn.00372.2016 27535374 PMC5110639

[B100] Profant O. Škoch A. Tintìra J. Svobodová V. Kuchárová D. Svobodová Burianová J. (2020). The influence of aging, hearing, and tinnitus on the morphology of cortical gray matter, amygdala, and hippocampus. *Front. Aging Neurosci.* 12:553461. 10.3389/fnagi.2020.553461 33343328 PMC7746808

[B101] Pronk M. Deeg D. J. Festen J. M. Twisk J. W. Smits C. Comijs H. C. (2013). Decline in older persons’ ability to recognize speech in noise: The influence of demographic, health-related, environmental, and cognitive factors. *Ear Hear.* 34 722–732. 10.1097/AUD.0b013e3182994eee 24165301

[B102] R Core Team (2021). *R: A language and environment for statistical computing.* Vienna: R Foundation for Statistical Computing.

[B103] Rabbitt P. (1993). Does it all go together when it goes? The nineteenth bartlett memorial lecture. *Q J Exp. Psychol. A* 46 385–434. 10.1080/14640749308401055 8378549

[B104] Reinhart P. N. Souza P. E. (2016). Intelligibility and clarity of reverberant speech: Effects of wide dynamic range compression release time and working memory. *J. Speech Lang. Hear. Res.* 59 1543–1554. 10.1044/2016_JSLHR-H-15-0371 27997667 PMC5399768

[B105] Reuter-Lorenz P. A. Jonides J. Smith E. E. Hartley A. Miller A. Marshuetz C. (2000). Age differences in the frontal lateralization of verbal and spatial working memory revealed by PET. *J. Cogn. Neurosci.* 12 174–187. 10.1162/089892900561814 10769314

[B106] Rönnberg J. Lunner T. Ng E. H. Lidestam B. Zekveld A. A. Sörqvist P. (2016). Hearing impairment, cognition and speech understanding: Exploratory factor analyses of a comprehensive test battery for a group of hearing aid users, the n200 study. *Int. J. Audiol.* 55 623–642. 10.1080/14992027.2016.1219775 27589015 PMC5044772

[B107] Rönnberg J. Lunner T. Zekveld A. Sörqvist P. Danielsson H. Lyxell B. (2013). The ease of language understanding (ELU) model: Theoretical, empirical, and clinical advances. *Front. Syst. Neurosci.* 7:31. 10.3389/fnsys.2013.00031 23874273 PMC3710434

[B108] Rosano C. Simonsick E. M. Harris T. B. Kritchevsky S. B. Brach J. Visser M. (2005). Association between physical and cognitive function in healthy elderly: The health, aging and body composition study. *Neuroepidemiology* 24 8–14. 10.1159/000081043 15459503

[B109] Salthouse T. A. (1996). The processing-speed theory of adult age differences in cognition. *Psychol. Rev.* 103 403–428. 8759042 10.1037/0033-295x.103.3.403

[B110] Salthouse T. A. (2009). When does age-related cognitive decline begin?. *Neurobiol. Aging.* 30 507–514. 10.1016/j.neurobiolaging.2008.09.023 19231028 PMC2683339

[B111] Salthouse T. A. (2019). Trajectories of normal cognitive aging. *Psychol. Aging* 34 17–24. 10.1037/pag0000288 30211596 PMC6367038

[B112] Scarpina F. Tagini S. (2017). The stroop color and word test. *Front. Psychol.* 8:557. 10.3389/fpsyg.2017.00557 28446889 PMC5388755

[B113] Schneider B. A. Pichora-Fuller M. K. (2000). “Implications of perceptual deterioration for cognitive aging research,” in *The handbook of aging and cognition*, eds Craik F. I. M. Salthouse T. A. (Mahwah, NJ: Lawrence Erlbau Associates), 155–219.

[B114] Schoene D. Wu S. M. Mikolaizak A. S. Menant J. C. Smith S. T. Delbaere K. (2013). Discriminative ability and predictive validity of the timed up and go test in identifying older people who fall: Systematic review and meta-analysis. *J. Am. Geriatr. Soc.* 61 202–208. 10.1111/jgs.12106 23350947

[B115] Sicard V. Moore R. D. Simard A. Lavoie G. Ellemberg D. (2020). Psychometric properties of a color-shape version of the switch task. *Appl. Neuropsychol. Adult* 9 1–10. 10.1080/23279095.2020.1842410 33297758

[B116] Singh G. Pichora-Fuller M. K. Schneider B. A. (2013). Time course and cost of misdirecting auditory spatial attention in younger and older adults. *Ear Hear.* 34 711–721. 10.1097/AUD.0b013e31829bf6ec 24165300

[B117] Smits C. Theo Goverts S. Festen J. M. (2013). The digits-in-noise test: Assessing auditory speech recognition abilities in noise. *J. Acoust. Soc. Am.* 133 1693–1706. 10.1121/1.4789933 23464039

[B118] Soumaré A. Tavernier B. Alpérovitch A. Tzourio C. Elbaz A. (2009). A cross-sectional and longitudinal study of the relationship between walking speed and cognitive function in community-dwelling elderly people. *J. Gerontol. A Biol. Sci. Med. Sci.* 64 1058–1065. 10.1093/gerona/glp077 19561146

[B119] Stephan D. N. Hensen S. Fintor E. Krampe R. Koch I. (2018). Influences of postural control on cognitive control in task switching. *Front. Psychol.* 5:1153. 10.3389/fpsyg.2018.01153 30344499 PMC6182063

[B120] Stevens J. C. Alvarez-Reeves M. Dipietro L. Mack G. W. Green G. (2003). Decline of tactile acuity in aging: A study of body site, blood flow, and lifetime habits of smoking and physical activity. *Somatosens Mot. Res.* 20 271–279. 10.1080/08990220310001622997 14675966

[B121] Strelcyk O. Zahorik P. Shehorn J. Patro C. Derleth R. P. (2019). Sensitivity to interaural phase in older hearing-impaired listeners correlates with nonauditory trail making scores and with a spatial auditory task of unrelated peripheral origin. *Trends Hear.* 23:2331216519864499. 10.1177/2331216519864499 31455167 PMC6755865

[B122] Strouse A. Ashmead D. H. Ohde R. N. Grantham D. W. (1998). Temporal processing in the aging auditory system. *J. Acoust. Soc. Am.* 104 2385–2399. 10.1121/1.423748 10491702

[B123] Sussman E. Winkler I. (2001). Dynamic sensory updating in the auditory system. *Brain Res. Cogn. Brain Res.* 12 431–439. 10.1016/s0926-6410(01)00067-2 11689303

[B124] Tinetti M. E. Speechley M. Ginter S. F. (1988). Risk factors for falls among elderly persons living in the community. *N. Engl. J. Med.* 319 1701–1707. 10.1056/nejm198812293192604 3205267

[B125] Tingley D. Yamamoto T. Hirose K. Keele L. Imai K. (2014). R package for causal mediation analysis. *J. Stat. Softw.* 59 1–38.26917999

[B126] Tun P. A. O’Kane G. Wingfield A. (2002). Distraction by competing speech in young and older adult listeners. *Psychol. Aging* 17 453–467. 10.1037//0882-7974.17.3.453 12243387

[B127] Uchida Y. Sugiura S. Nishita Y. Saji N. Sone M. Ueda H. (2019). Age-related hearing loss and cognitive decline–The potential mechanisms linking the two. *Auris Nasus Larynx* 46 1–9. 10.1016/j.anl.2018.08.010 30177417

[B128] Vercammen C. Goossens T. Undurraga J. Wouters J. van Wieringen A. (2018b). Electrophysiological and behavioral evidence of reduced binaural temporal processing in the aging and hearing impaired human auditory system. *Trends Hear.* 22:2331216518785733. 10.1177/2331216518785733 30022734 PMC6053861

[B129] Vercammen C. Goossens T. Wouters J. van Wieringen A. (2018a). DTT hearing screening with broadband and low-pass filtered noise in a middle-aged population. *Ear Hear.* 39 825–882. 10.1097/AUD.0000000000000524 29189521

[B130] Vereeck L. Wuyts F. Truijen S. Van de Heyning P. (2008). Clinical assessment of balance: Normative data, and gender and age effects. *Int. J. Audiol.* 47 67–75. 10.1080/14992020701689688 18236239

[B131] Verhaeghen P. Cerella J. (2002). Aging, executive control, and attention: A review of meta-analyses. *Neurosci. Biobehav. Rev.* 26 849–857. 10.1016/s0149-7634(02)00071-4 12470697

[B132] Wechsler D. (2001). *WAIS-III Nederlandstalige bewerking. Afname en Scoringshandleiding.* Lisse: Swets & Zeitlinger.

[B133] Wechsler D. (2008). *Wechsler adult intelligence scale—fourth edition (WAIS–IV).* San Antonio, TX: NCS Pearson.

[B134] Wells C. Ward L. M. Chua R. Inglis J. T. (2003). Regional variation and changes with ageing in vibrotactile sensitivity in the human footsole. *J. Gerontol. A Biol. Sci. Med. Sci.* 58 680–686. 10.1093/gerona/58.8.b680 12902525

[B135] Wingfield A. (1996). Cognitive factors in auditory performance: Context, speed of processing, and constraints of memory. *J. Am. Acad. Audiol.* 7 175–182. 8780990

[B136] Yogev-Seligmann G. Hausdorff J. M. Giladi N. (2008). The role of executive function and attention in gait. *Mov. Disord.* 23 329–342. 10.1002/mds.21720 18058946 PMC2535903

[B137] Ziso B. Larner A. J. (2019). Codex (cognitive disorders examination) decision tree modified for the detection of dementia and MCI. *Diagnostics (Basel)* 9:58. 10.3390/diagnostics9020058 31159432 PMC6628135

