# Supplementary material for: Coupling of sensorimotor and cognitive functions in middle- and late adulthood
Source: Front Neurosci. 2022 Dec 1;16:1049639. doi: 10.3389/fnins.2022.1049639 (PMC9752872; doi:10.3389/fnins.2022.1049639)
Supplement: Supplementary file 1 [file Data_Sheet_1.pdf]

Supplementary files

Info participants

	46-55	56-65	66-75	76-86
Smoked for more than 1 year	8	10	12	5
Wears one or two hearing aids	0	1	2	3
Fallen within the last year	1	3	2	1
Hip or knee prosthesis	0	1	3	2
Followed physical therapy	1	3	5	7
Reported a (previous) ear disease	1	1	4	3

Full regression models

			Processing speed	Cognitive Control		
				Task Switching	Inhibition	Working Memory Updating
		Predictors	Estimate (CI)	Estimate (CI)	Estimate (CI)	Estimate (CI)
Listening in Noise	DiN	Age group *‘MA’* vs. *’O’*	0.32 (0.31-0.33) ***	0.32 (0.31-0.33) ***	0.32 (0.31-0.33) ***	0.32 (0.31-0.33) ***
		Age group *‘YO’* vs. *’OO’*	0.19 (0.15-0.23) ***	0.19 (0.15-0.23) ***	0.19 (0.15-0.23) ***	0.19 (0.15-0.23) ***
		Age group *‘YMA’* vs. *’OMA’’*	0.05 (0.02-0.08) ***	0.05 (0.02-0.08) ***	0.05 (0.02-0.08) ***	0.05 (0.02-0.08) ***
		Median split *‘low vs. high performance’*				
		Median split x age group *‘MA’* vs. *’O’*				
		Median split x age group *‘YO’* vs. *’OO’*	-0.04 (0.02-0.08) ***			
		Median split x age group *‘YMA’* vs. *’OMA’’*				
			R^2= 0.6 , R_{adj}^2= 0.57	R^2=0.56 , R_{adj}^2=0.53	R^2=0.55 , R_{adj}^2=0.52	R^2=0.54 , R_{adj}^2=0.50
Functional Mobility	TUG	Age group *‘MA’* vs. *’O’*	0.03 (0.02-0.03)***	0.03 (0.02-0.03)***	0.03 (0.02-0.03)***	0.03 (0.02-0.03)***
		Age group *‘YO’* vs. *’OO’*	0.01 (0.00-0.01)*	0.01 (0.00-0.01)*	0.01 (0.00-0.01)*	0.01 (0.00-0.01)*
		Age group *‘YMA’* vs. *’OMA’’*	0.01 (0.00-0.02)*	0.01 (0.00-0.02)*	0.01 (0.00-0.02)*	0.01 (0.00-0.02)*
		Median split *‘low vs. high performance’*	-0.00 (-0.00--0.00) *	-0.00 (-0.01-0.00)***	-0.00 (-0.01--0.00)**	-0.00 (-0.00—0.00)**
		Median split x age group *‘MA’* vs. *’O’*	-0.01 (-0.02-0.00)*			-0.01 (0.02—0.00)**
		Median split x age group *‘YO’* vs. *’OO’*				
		Median split x age group *‘YMA’* vs. *’OMA’*	-0.01 (0.01—0.00)**			
			R^2=0.48 , R_{adj}^2=0.43	R^2=0.43 , R_{adj}^2=0.39	R^2=0.44 , R_{adj}^2=0.40	R^2= 0.42, R_{adj}^2=0.37
Postural Control	Short-term Diffusion Coefficient	Age group *‘MA’* vs. *’O’*	0.59 (0.37-0.81)***	0.59 (0.37-0.81)***	0.59 (0.37-0.81)***	0.59 (0.37-0.81)***
		Age group *‘YO’* vs. *’OO’*				
		Age group *‘YMA’* vs. *’OMA’’*				
		Median split effect *‘low vs. high performance’*				
		Median split x age group *‘MA’* vs. *’O’*	-0.24 (-0.46—0.02)*			
		Median split x age group *‘YO’* vs. *’OO’*	-0.17 (-0.32—0.01)*			
		Median split x age group *‘YMA’* vs. *’OMA’*				
			R^2=0.34 , R_{adj}^2=0.29	R^2=0.27 , R_{adj}^2=0.21	R^2=0.24 , R_{adj}^2=0.18	R^2=0.26 , R_{adj}^2=0.20
